# A prognostic gene expression signature for oropharyngeal squamous cell carcinoma

**DOI:** 10.1016/j.ebiom.2020.102805

**Published:** 2020-10-07

**Authors:** Xinyi Liu, Ping Liu, Rebecca D. Chernock, Krystle A.Lang Kuhs, James S. Lewis, Jingqin Luo, Hiram A. Gay, Wade L. Thorstad, Xiaowei Wang

**Affiliations:** a Department of Radiation Oncology, Washington University School of Medicine, St. Louis, MO, USA; b Department of Pathology and Immunology, Washington University School of Medicine, St. Louis, MO, USA; c Department of Surgery, Washington University School of Medicine, St. Louis, MO, USA; d Department of Otolaryngology, Microbiology and Immunology, Vanderbilt University Medical Center, Nashville, TN, USA; e Department of Pathology, Microbiology and Immunology, Vanderbilt University Medical Center, Nashville, TN, USA

**Keywords:** Oropharyngeal cancer, Prognosis, Gene signature, RNA-seq

## Abstract

**Background:**

Robust prognostic stratification of patients with oropharyngeal squamous cell carcinoma (OPSCC) is important for developing individualized treatment plans. This study was conducted to develop and validate a clinically feasible prognostic classifier based on transcriptome-wide gene expression profiles.

**Methods:**

Tumor tissues were collected from 208 OPSCC patients treated at Washington University in St. Louis and 130 OPSCC patients treated at Vanderbilt University, used for model training and validation, respectively. OPSCC patients (*n* = 70) from the TCGA cohort were also included for independent validation. Based on RNA-seq profiling data, Cox proportional hazards regression analysis was performed to identify genes associated with disease outcomes. Then, Lasso-penalized multivariate survival models were constructed to identify biomarker genes for developing a prognostic gene signature.

**Findings:**

A 60-gene signature was identified by RNA-seq profiling analysis. Computed risk score of the gene signature was significantly predictive of 5-year overall survival of the training cohort (Hazard ratio (HR) 28·32, *P* = 4·3E-41). Subgroup analysis stratified by HPV status revealed that the signature was prognostic in HPV-positive OPSCC patients (HR 30·55, *P* = 7·0E-37) and was independent of clinical features. Importantly, the gene signature was validated in two independent patient cohorts, including the TCGA cohort (HR 3·94, *P* = 0·0018) and the Vanderbilt cohort (HR 8·50, *P* = 5·7E-09) for overall survival.

**Interpretation:**

The prognostic gene signature is a robust tool for risk stratification of OPSCC patients. The signature remains prognostic among HPV-positive OPSCC patients.

**Funding:**

National Institutes of Health.

**Research in context**

*Evidence before this study*

Accurate prognostication of patients with oropharyngeal squamous cell carcinoma (OPSCC) is critical for providing effective individualized treatment plans. Although HPV-positive (HPV+) patients show better overall survival, there are approximately 20% of HPV+ OPSCC having dismal prognosis. We systematically searched PubMed up to September 15, 2019, with the search terms “mRNA”, “OPSCC”, “prognostic”, and “biomarker”, with no publication date or language restrictions. Of the OPSCC mRNA signature studies, several signatures were developed by analyzing small numbers of genes using limited numbers of tumor specimens. For example, high expression level of HER3 was found to be significantly correlated with poor overall survival (OS); patients with highly expressed nuclear PRMT5 had 1·7 times higher hazard of death; an immune-related gene signature was reported to be prognostic of OS; a hypoxia-related gene signature was reported to be associated with poor progression-free survival; an HPV-correlated signature including 38 genes was prognostic of HPV+ OPSCC patients. Our search did not identify any previous transcriptome level studies that investigated the potential predictive role of mRNA expression signature in OPSCC.

*Added value of this study*

We did a cross-institutional study to develop an mRNA expression signature for predicting the risk of OPSCC progression. By performing transcriptome-wide RNA-seq profiling analysis for 408 OPSCC cases, a 60-gene signature was developed. Computed risk score of the gene signature was significantly predictive of 5-year overall, 5-year recurrence-free, and 5-year metastasis-free survival of the training cohort. Subgroup analysis stratified by HPV status revealed that the signature was prognostic in HPV+ OPSCC patients. We validated the performance of the 60-gene signature in two independent cohorts and the signature maintained its independent prognostic value after adjustment for the clinical variables. Our finding suggests that the 60-gene signature is a clinically feasible signature for OPSCC prognosis including the HPV+ OPSCC subgroup.

*Implications of all the available evidence*

We developed and validated a clinically feasible gene signature for the prognosis of OPSCC patients including the subgroup of HPV+ patients. The robust performance of the signature on multi-institutional OPSCC patients indicates its general applicability in clinical practice.

Alt-text: Unlabelled box

## 1 Introduction

Epidemiologic evidence has shown a rapid increase in the incidence rate of oropharyngeal squamous cell carcinoma (OPSCC) in the past several decades [1]. Accurate prognostication of patients with OPSCC is critical for providing effective individualized treatment plans. Currently, OPSCC prognosis is mainly based on human papillomavirus (HPV) status, lifetime tobacco cigarette smoking and tumor and nodal stage [2]. Although HPV-positive (HPV+) patients show better overall survival, disease outcomes still vary greatly, and rates of distant metastasis are similar to the HPV-negative (HPV-) population [3].

Multiple prognostic models for OPSCC have been developed in recent years based on specific domain knowledge of small subsets of genes, such as those focused on specific somatic mutations [4], HPV-promoter methylome [5], miRNA [6] and immune-related genes [7]. However, knowledge of the molecular mechanisms underlying observed clinical differences remains rather limited at present, and existing prognostic models have not been validated in large independent OPSCC patient population. Thus, there is an urgent need to identify robust prognostic biomarkers which further stratify risk in OPSCC patients.

In general, an integrated model consisting of multiple genes have greater predictive ability than single gene models [8]. To this end, traditional univariate or multivariate Cox regression models are commonly used to select biomarker genes for further building multi-gene signatures. However, conventional Cox regression models, without adequate consideration of variable selection, often suffer from increased risk of model overfitting when a large number of genes are included. To address this challenge, the least absolute shrinkage and selection operator (LASSO) [9] penalized Cox model is a useful method to implement variable selection while fitting the Cox model and has been widely applied for robust modeling of high-dimensional predictors. In this study, we sought to develop a robust prognostic gene signature for OPSCC based on transcriptional expression profiles of genes, as selected by LASSO Cox regression models. A patient cohort from Washington University in St. Louis was used as the training set to build the prognostic signature. An independent OPSCC cohort from Vanderbilt University as well as an OPSCC cohort from The Cancer Genome Atlas (TCGA) [10] were used to evaluate the general applicability of the signature. In this way, we have developed and validated an RNA expression based 60-gene signature that robustly predicted OPSCC survival across multiple institutions.

## 2 Methods

### 2.1 Patients cohorts

This study was approved by the Human Research Protection Office of the Washington University in St. Louis and the Human Research Protection Program of Vanderbilt University Medical Center. Informed patient consent was not required for this retrospective study. Two hundred and eight OPSCC cases treated at Washington University were included in this study as the training cohort. In addition, we obtained 130 cases treated at Vanderbilt University as a validation cohort. Clinical data were collected prospectively from these patients and then updated retrospectively after follow-up review.

For all the patients, formalin-fixed, paraffin-embedded (FFPE) tumor tissues were collected for pathologic analysis from biopsy or primary surgical resection. Tumor sections from these cases were stained with hematoxylin and eosin (H&E) and reviewed independently by pathologists at Washington University and Vanderbilt University to confirm the diagnoses. Tumor regions from each section were identified by H&E staining, and total RNA was then extracted from macrodissected tumor regions with the miRNeasy FFPE kit (Qiagen). In this way, we focused on expression profiling of the tumor tissues with minimal contamination from adjacent normal tissues.

From the TCGA GDC Data Portal (https://gdc-portal.nci.nih.gov/), normalized RNA-seq and clinical data for head and neck cancer (HNSCC) patients were retrieved. From this cohort, a total of 70 OPSCC patients were identified according to anatomic neoplasm subdivision annotations in the clinical data.

### 2.2 RNA-seq and sequencing data analysis

Details of the RNA-seq experimental protocol have been described previously [11]. In brief, ribosomal RNA (rRNA) was removed with the RiboMinus kit (Life Technologies) and custom DNA oligonucleotide probes. Then, the RNA was used to construct RNA-seq libraries with the NEBNext Ultra mRNA Library Prep kit (New England BioLabs). The resulting cDNA libraries were PCR amplified and subject to sequencing with Illumina HiSeq 3000 at Washington University Genome Technology Access Center. Raw sequencing reads were mapped to the NCBI reference sequence database [12] to identify human coding genes as well as aligned to annotated HPV genomes [13] to identify HPV transcripts with Bowtie [14]. Twenty-seven samples were excluded from a total of 338 samples due to low sequencing data quality (i.e. total mRNA read counts < 400,000). The read counts were normalized to reads per kilobase per million reads (RPKM). Genes with average RPKM <1 were excluded from further analysis. The RPKM expression values were further log2 transformed.

### 2.3 Construction of gene expression prediction model

Univariate Cox regression analysis was performed to evaluate the association between individual gene expression and disease outcome in the training data. A simple mean imputation approach was used to replace missing values in the clinical variables. The average percentage of missing values is 2.4% per clinical variable in the training cohort and 2.2% in the Vanderbilt validation cohort. Multivariate Cox regression analysis was performed to evaluate the independent prognostic value of individual genes after controlling for common clinicopathologic variables. LASSO was used to reduce model overfitting during the selection of high-performance biomarker genes with the glmnet R Package [15]. Specifically, an optimal value for the penalty parameter (λ1) was calculated using a ten-fold internal cross-validation for selecting variables in the final model. The modeling process was repeated 1000 times. From the 1000 iterations, different gene models were constructed for optimal prognostic performance. We then counted the occurrence of individual genes across all presented models. Various cutoffs were considered to retain genes with occurrences >100, 200 or 300 to develop three respective gene signatures. The risk score of the signatures was calculated as follows:

$$\text{Risk}\;\text{score}=\sum_{i=1}^{p}\left(z_{i}*e_{i}\right)$$

where *p* is the total number of genes retained in the final signature, *i* indicates the *i*th gene in the signature, *z_i_* and *e_i_* represent the z-score (i.e., the Cox regression coefficient of a gene divided by its estimated standard error) and the expression level of the *i*th gene, respectively. C-index from the Cox analysis was applied to evaluate the performance of the signatures. Patient stratification according to risk score was done using the optimal cut-off value as established in the training cohort by receiver operating characteristic (ROC) curve analysis for overall survival.

### 2.4 Statistical analysis

In our study, overall survival (OS) was defined as the time interval between the date of diagnosis and the date of death; recurrence-free survival (RFS) was defined as the time interval between the date of diagnosis and the date of first recurrence or death; metastasis-free survival (MFS) was defined as the time interval between the date of diagnosis and the date of first distant metastasis or death. The Kaplan-Meier survival curve and log-rank test were used to evaluate the performance of the gene signatures. Statistical data analysis was performed using the statistical programming language R (http://www.r-project.org/).

## 3 Results

### 3.1 RNA-seq profiling to globally identify genes associated with patient survival

RNA-seq profiling was performed for 208 OPSCC patients treated at Washington University in St. Louis. Total RNA isolated from FFPE tumor specimens is usually highly degraded, and thus not suitable for conventional RNA-seq profiling analysis. To address this challenge, we developed a sequencing library construction method using custom designed probes for rRNA removal (see Methods for details). In this way, most rRNA reads were successfully removed from the constructed libraries. On average, we obtained 12 million total reads per sample. After removing 11 samples with low sequencing data quality, 197 cases were included in the training cohort for discovering prognostic biomarker genes (Table 1). Among them, 32.5% (*n* = 64) of the patients received surgery with adjuvant chemoradiation therapy, 35.0% (*n* = 69) received surgery with adjuvant radiation therapy, 16.2% (*n* = 32) received chemoradiation therapy and 11.2% (*n* = 22) received surgery only. The median follow-up time was 60 months, with 49 of the 197 patients (24·9%) deceased during the study period. We performed univariate Cox regression analysis to identify genes whose RNA expression was correlated with 5-year (5-yr) overall survival in the training cohort. The results revealed that 1928 protein-coding genes were associated with 5-yr OS (*P* < 0·05, Wald test). The independent prognostic values of these genes were evaluated further by controlling for treatment protocols (chemotherapy and radiotherapy status) with multivariate Cox regression analysis. In this way, 1436 genes were selected as biomarker candidates for further model development (*P* < 0·05, Wald test).

**Table 1:** Patient characteristics.

Variable	WashU Cohort (*n* = 197)	Vanderbilt Cohort (*n* = 114)	TCGA Cohort (*n* = 70)
**Median age (IQR)**	57 (50–64)	58 (51–64)	56 (50–62)
**Sex, male (%)**	172 (87·3)	100 (87·7)	60 (85·7)
**Race (%)**			
White	184 (93·4)	107 (93·9)	65 (92·9)
Other	13 (6·6)	7 (6·1)	5 (7·1)
**HPV status (%)**			
Negative	30 (15·2)	21 (18·4)	26 (37·1)
Positive	167 (84·8)	93 (81·6)	44 (62·9)
**Stage (%)**			
Stage I–II	13 (6·6)	17 (14·9)	15 (21·4)
Stage III–IV	174 (88·3)	80 (70·2)	53 (75·7)
**Smoking (%)**			
Non-smoker	62 (31·5)	30 (26·3)	19 (27·1)
Smoker	131 (66·5)	70 (61·4)	50 (71·4)
**5-year events (%)**			
Recurrence	31 (15·7)	19 (16·7)	9 (12·9)
Death	49 (24·9)	27 (23·7)	20 (28·6)

### 3.2 A gene expression signature to predict patient survival

To further identify the most promising gene biomarkers, we performed LASSO-penalized multivariate Cox modeling with ten-fold cross-validation. Across 1000 iterations, 16 different gene expression models were constructed for optimal survival prediction. The frequency counts of these different models ranged from 1 to 681. A total of 77 genes were represented in all the models combined, with a range of 6 to 59 genes in individual models. The frequency counts of the 77 genes were in the range of 5 to 700 across 1000 iterations (Supplementary Table S1). We established three gene signatures based on various cutoffs of the gene occurrence (>100, 200 or 300). We found that the signature consisting of 60 genes (*n*>200) had the best prognostic performance (C-index = 0·89). Thus, this signature was selected for further analysis. Among the 60 genes in this signature, 26 had positive Cox coefficients, indicating that higher expression levels were associated with poor survival outcomes. On the other hand, 34 genes had negative Cox coefficients, indicating higher expression levels were associated with favorable outcomes (Supplementary Table S2).

**Fig. 1:**
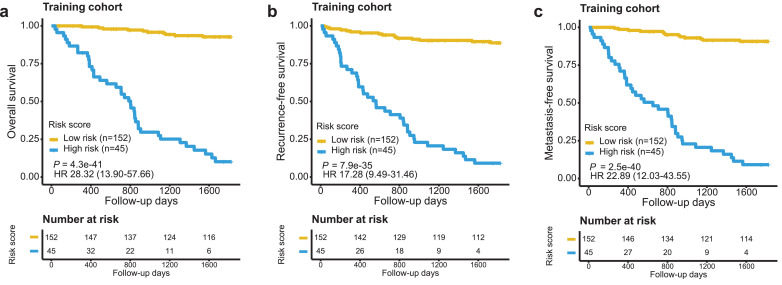
Evaluation of the 60-gene signature for 5-yr OS **(a)**, 5-yr RFS **(b)** and 5-yr MFS **(c)** in the training cohort. Patients were stratified according to signature risk score. Kaplan-Meier survival analysis was performed, with log-rank *P* values presented.

A risk scoring system was established by summarizing weighted expression values of the 60 genes (see Methods). The z-scores in the univariate Cox models were used as weighting factors to compute the risk score. In this way, we calculated the risk score for each patient in the training cohort. ROC analysis was performed to define the optimal cut-off for patient risk stratification, revealing a sensitivity of 80% and a specificity of 96% at the cut-off value of −158·3 (Supplementary Figure S1). Patients were divided into high-risk and low-risk groups based on the optimal cut-off value. In this way, 53 patients were predicted to be of high-risk (with score >−158·3) and 144 of low-risk (with score < −158·3). Expression heatmap analysis indicates that the 34 favorable and 26 unfavorable genes had enriched expression in patients with low- and high-risk scores, respectively (Supplementary Figure S2). In total, 86·7% of the patients in the high-risk group vs. 6·6% in the low-risk group died during the study period. Kaplan-Meier survival analysis indicated that the death risk for high-risk patients was significantly higher compared with that for low-risk patients (HR 28·32, 95% CI 13·90–57·66, *P* = 4·3E-41, Log-rank test; Fig. 1a). Moreover, the risk score was also demonstrated to be prognostic of locoregional and distant failures (HR 17·28, 95% CI 9·49–31·46, *P* = 7·9E-35 for 5-y RFS and HR 22·89, 95% CI 12·03–43·55, *P* = 2·5E-40 for 5-y MFS, Log-rank test; Fig. 1b and c).

### 3.3 The 60-gene signature was independent of clinicopathologic features and HPV status

We assessed whether the 60-gene signature had independent prognostic value in the context of clinicopathologic parameters including age, sex, race, smoking history, TNM stage, radiotherapy and chemotherapy status. With univariate Cox analysis, the 60-gene signature were significantly associated with 5-yr OS in the training cohort (*P* = 3·1E-20, Wald test, Table 2). Multivariate Cox analysis further exhibited that the signature retained its independent prognostic value for 5-yr OS after adjusting for clinicopathologic factors (HR 30·19, 95% CI 14·41–63·23, *P* = 9·2E-16, Wald test; Table 2).

**Table 2:** Prognostic performance of the 60-gene signature in the context of clinicopathologic features in the training cohort.

Overall Survival Variable	Univariate Cox		Multivariate Cox	
	HR (95% CI)	*P* value	HR (95% CI)	*P* value
**Training cohort (*n*** **=** **197)**				
Gene signature (low- vs high-risk)	28·32 (13·90–57·66)	3·1e-20*	30·19 (14·41–63·23)	9·2e-16*
Age	1·03 (1·00–1·06)	0·045*	1·03 (1·00–1·07)	0·05
Stage (I/II/III vs IV)	1·02 (0·52–2·00)	0·95	1·44 (0·69–3·00)	0·33
Sex (female vs male)	1·54 (0·55–4·28)	0·41	0·97 (0·30–3·18)	0·96
Race (white vs others)	1·76 (0·57–4·43)	0·23	0·98 (0·37–2·61)	0·97
Smoking (yes vs no)	0·59 (0·30–1·15)	0·12	1·16 (0·55–2·46)	0·69
Chemotherapy (no vs yes)	1·30 (0·74–2·30)	0·36	0·96 (0·48–1·92)	0·90
Radiotherapy (no vs yes)	1·51 (0·54–4·26)	0·44	1·19 (0·31–4·51)	0·80
**Training HPV+ cohort (*n*** **=** **167)**				
Gene signature (low- vs high-risk)	30·55 (13·86–67·34)	2·3e-17*	30·82 (13·01–72·98)	6·5e-15*
Age	1·05 (1·01–1·09)	7·7e-03*	1·02 (0·98–1·07)	0·24
Stage (I/II/III vs IV)	1·31 (0·55–3·11)	0·55	1·32 (0·45–3·84)	0·61
Sex (female vs male)	4·36 (0·60–31·93)	0·15	3·42 (0·35–33·64)	0·29
Race (white vs others)	0·96 (0·13–7·04)	0·97	3·00 (0·37–24·32)	0·30
Smoking (yes vs no)	0·66 (0·30–1·44)	0·29	1·11 (0·46–2·67)	0·82
Chemotherapy (no vs yes)	1·51 (0·74–3·06)	0·26	0·96 (0·38–2·43)	0·94
Radiotherapy (no vs yes)	1·44 (0·43–4·81)	0·56	0·90 (0·20–4·11)	0·89

⁎ *P* value (Wald test) is significant.

**Fig. 2:**
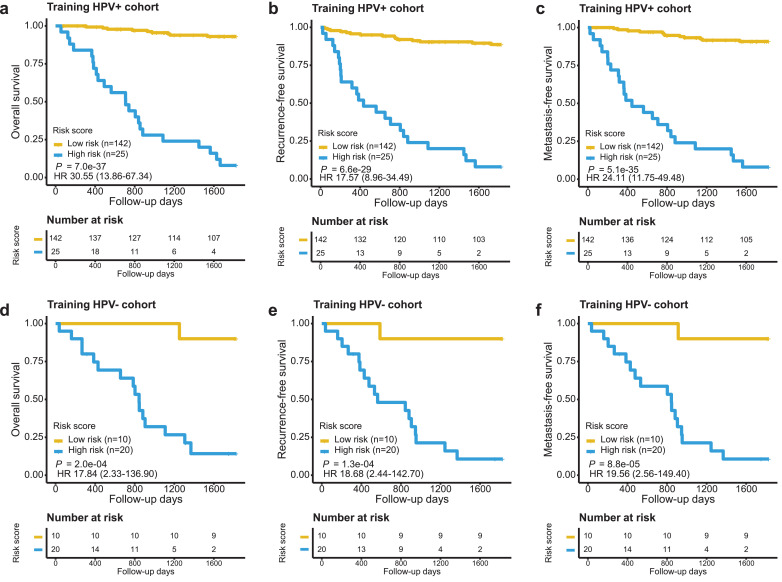
Evaluation of the 60-gene signature in the context of HPV status of the training cohort. The 5-yr OS, 5-yr RFS and 5-yr MFS were evaluated in the HPV+ patient group **(a-c)** and HPV- patient group **(d-f)**, respectively. Patients were stratified according to signature risk score. Kaplan-Meier survival analysis was performed, with log-rank *P* values presented.

It is well-known that HPV+ OPSCC is associated with better outcome than HPV- OPSCC. Given the strong prognostic impact of the HPV status on survival, we further assessed whether the 60-gene signature could significantly prognosticate both HPV+ and HPV- patients in the training cohort. The HPV status was determined by RNA-seq analysis as described in Methods. Patients were stratified into two groups according to their HPV status (167 HPV+ cases and 30 HPV- cases). The 60-gene signature was assessed for its performance by applying to these two patient subgroups separately. Survival analysis indicated that the signature risk score was prognostic of 5-yr OS (HR 30·55, 95% CI 13·86–67·34, *P* = 7·0E-37, Log-rank test), 5-yr RFS (HR 17·57, 95% CI 8·96–34·49, *P* = 6·6E-29, Log-rank test) and 5-yr MFS (HR 24·11, 95% CI 11·75–49·48, *P* = 5·1E-35, Log-rank test; Fig. 2a-c) for HPV+ patients. Similarly, the risk score was also a prognostic predictor for HPV- patients, although with less significant *p*-values (*p*-values for survival outcomes ranging from 8·8E-05 to 2·0E-04, Log-rank test) as compared with HPV+ patients, partly due to the smaller sample size (Fig. 2d-f). Importantly, the 60-gene signature also maintained its independent prognostic value in the HPV+ patient subgroup as evaluated by both univariate analysis (HR 30·55, 95% CI 13·86–67·34, *P* = 2·3E-17, Wald test) and multivariable analysis adjusting for clinical features (HR 30·82, 95% CI 13·01–72·98, *P* = 6·5E-15, Wald test; Table 2). Expression heatmap analysis indicates that the favorable and unfavorable genes in the signature had enriched expression in HPV+ OPSCCs with low- and high-risk scores, respectively (Supplementary Figure S3). Thus, the prognostic value of the 60-gene signature was independent of both the clinicopathologic features and HPV status.

### 3.4 Validation of the 60-gene signature in independent cross-institutional cohorts

**Fig. 3:**
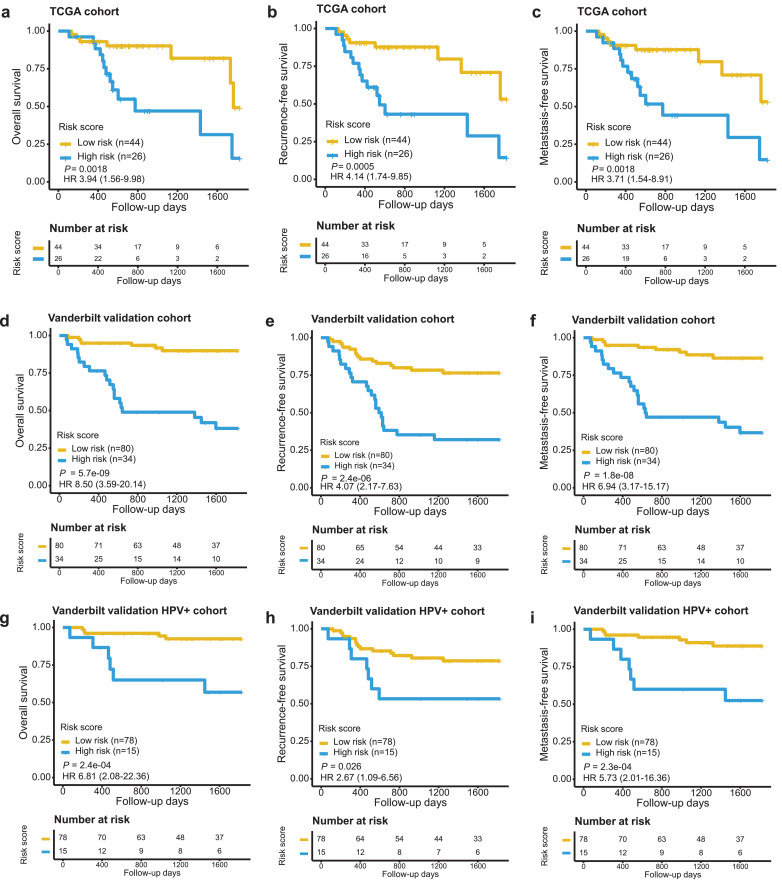
Evaluation of the 60-gene signature in the validation cohorts. The 5-yr OS, 5-yr RFS and 5-yr MFS in the TCGA validation cohort (**a-c**), Vanderbilt validation cohort (**d-f**) and Vanderbilt HPV+ subgroup **(g-i)** are presented. Patients were stratified according to signature risk score. Kaplan-Meier survival analysis was performed, with log-rank *P* values presented.

To assess the general applicability of the 60-gene signature, we first applied it to the TCGA OPSCC subset comprised of 70 patients (Table 1). A risk score was calculated for each patient. The same cut-off score value, as determined with the training cohort, was used for patient stratification. The patients with high-risk score (*n* = 26) had significantly shorter 5-yr OS (HR 3·94, 95% CI 1·56–9·98, *P* = 0·0018, Log-rank test), 5-yr RFS (HR 4·14, 95% CI 1·74–9·85, *P* = 0·0005, Log-rank test) and 5-yr MFS (HR 3·71, 95% CI 1·54–8·91, *P* = 0·0018, Log-rank test) than those with low-risk score (*n* = 44, Fig. 3a-c).

To more comprehensively evaluate the signature performance, we included an independent OPSCC cohort comprised of 130 patients treated at Vanderbilt University. Sixteen samples were removed due to the low sequencing data quality. Among the 114 patients, 38.6% (*n* = 44) received surgery with adjuvant chemoradiation therapy, 37.7% (*n* = 43) received chemoradiation therapy and 11.4% (*n* = 13) received surgery only. Using the gene signature with the same established cut-off value, the patients were stratified into either the high-risk group (37 patients) or the low-risk group (77 patients). We observed that 58·8% of the patients in the high-risk group and 8·8% in the low-risk group deceased, respectively. Kaplan-Meier survival analysis confirmed the prognostic significance of the 60-gene signature for 5-yr OS (HR 8·50, 95% CI 3·59–20·14, *P* = 5·7E-09, Log-rank test), 5-yr RFS (HR 4·07, 95% CI 2·17–7·63, *P* = 2·4E-06, Log-rank test) and 5-yr MFS (HR 6·94, 95% CI 3·17–15·17, *P* = 1·8E-08, Log-rank test; Fig. 3d–f).

After adjusting for the clinicopathologic parameters in multivariate Cox regression analysis, the 60-gene signature retained its independent prognostic value in both validation cohorts (HR 3·46, 95% CI 1·21–9·85, *P* = 0·02 for the TCGA cohort and HR 5·53, 95% CI 2·17–14·09, *P* = 3·4E-04 for the Vanderbilt cohort, Wald test; Table 3). We also evaluated the prognostic power of the signature in the context of the HPV status. It was not feasible to risk-stratify TCGA HPV+ patients mainly due to their uniformly favorable outcomes as well as the relatively small sample size (*n* = 44). As to the Vanderbilt cohort, the risk score was prognostic of 5-yr OS when applied to the 93 HPV+ cases (HR 6·81, 95% CI 2·08–22·36, *P* = 2·4E-04, Log-rank test). Similarly, the risk score was also prognostic of 5-yr RFS (HR 2·67, 95% CI 1·09–6·56, *P* = 0·026, Log-rank test) and 5-yr MFS (HR 5·73, 95% CI 2·01–16·36, *P* = 2·3E-04, Log-rank test) for HPV+ patients (Fig. 3g-i). Importantly, multivariate Cox analysis showed that the 60-gene signature was an independent prognostic factor associated with 5-yr OS for HPV+ patients (HR 5·00, 95% CI 1·40–17·85, *P* = 0·01, Wald test; Table 3). As to the 21 HPV- patients, all except three were predicted to have high risk by the gene signature. In summary, the 60-gene signature retained its independent prognostic significance when applied to cross-institutional patient cohorts, including the subgroup of HPV+ patients.

**Table 3:** Prognostic performance of the 60-gene signature in the context of clinicopathologic features in the validation cohorts.

Overall Survival Variable	Univariate Cox		Multivariate Cox	
	HR (95% CI)	*P* value	HR (95% CI)	*P* value
**Vanderbilt validation cohort (*n*** **=** **114)**				
Gene signature (low- vs high-risk)	8·50 (3·59–20·14)	1·2e-06*	5·53 (2·17–14·09)	3·4e-04*
Age	1·09 (1·04–1·14)	2·6e-04*	1·06 (1·00–1·11)	0·05
Stage (I/II/III vs IV)	0·80 (0·34–1·88)	0·61	1·42 (0·52–3·92)	0·49
Sex (female vs male)	0·55 (0·21–1·45)	0·23	1·11 (0·33–3·76)	0·87
Race (white vs others)	2·90 (1·00–8·39)	0·05	1·83 (0·55–6·12)	0·33
Smoking (yes vs no)	0·18 (0·05–0·70)	0·01*	0·29 (0·07–1·25)	0·10
Chemotherapy (no vs yes)	0·68 (0·28–1·67)	0·40	0·53 (0·06–4·41)	0·56
Radiotherapy (no vs yes)	0·58 (0·22–1·50)	0·26	1·62 (0·17–15·18)	0·67
**Vanderbilt validation HPV+ cohort (*n*** **=** **93)**				
Gene signature (low- vs high-risk)	6·81 (2·08–22·36)	1·6e-03*	5·00 (1·40–17·85)	0·01*
Age	1·09 (1·00–1·17)	0·03*	1·09 (1·00–1·18)	0·049*
Stage (I/II/III vs IV)	0·56 (0·14–2·19)	0·40	0·28 (0·07–1·22)	0·09
Sex (female vs male)	NA	NA	NA	NA
Race (white vs others)	NA	NA	NA	NA
Smoking (yes vs no)	0·20 (0·03–1·28)	0·09	0·30 (0·04–2·01)	0·21
Chemotherapy (no vs yes)	10·21 (0·11–963·1)	0·32	NA	NA
Radiotherapy (no vs yes)	6·66 (0·07–631·2)	0·40	NA	NA
**TCGA validation cohort (*n*** **=** **70)**				
Gene signature (low- vs high-risk)	3·94 (1·56–9·98)	3·8e-03*	3·46 (1·21–9·85)	0·02*
Age	1·04 (0·99–1·09)	0·11	1·03 (0·98–1·08)	0·27
Stage (I/II/III vs IV)	0·66 (0·27–1·62)	0·36	0·94 (0·31–2·86)	0·92
Sex (female vs male)	0·45 (0·16–1·28)	0·14	0·55 (0·17–1·76)	0·31
Race (white vs others)	1·43 (0·19–11·00)	0·73	0·74 (0·08–6·42)	0·78
Smoking (yes vs no)	0·27 (0·08–0·94)	0·04*	0·41 (0·10–1·69)	0·22
Chemotherapy (no vs yes)	1·14 (0·43–2·98)	0·79	1·14 (0·31–4·20)	0·85
Radiotherapy (no vs yes)	0·74 (0·28–1·93)	0·54	1·14 (0·31–4·22)	0·84

⁎ *P* value (Wald test) is significant.

## 3 Discussion

In this study, we developed a comprehensive gene expression signature for OPSCC prognosis by RNA-seq transcriptome profiling. Our analysis indicated that the signature was independent from the clinical parameters as well as HPV status. Previous studies have identified multiple prognostic signatures for the general HNSCC population. However, signatures developed in this way may not perform well for OPSCC patients as OPSCC is distinctively different from most other subtypes of HNSCC. Attempting to address this challenge, several prognostic gene signatures were developed specifically for OPSCCs [16–18]. For example, high expression level of HER3 was found to be significantly correlated with poor OS [16]; patients with highly expressed nuclear PRMT5 had 1·7 times higher hazard of death [17]; an immune-related gene signature was reported to be prognostic of OS [7]; a hypoxia-related gene signature was reported to be associated with poor progression-free survival [18]; an HPV-correlated signature including 38 genes was prognostic of OPSCC patients [19]. However, these existing signatures were developed by analyzing small numbers of genes using limited numbers of tumor specimens. In contrast, the present study generated transcriptome-wide RNA-seq profiling data for over 300 OPSCC patients treated at two institutions. To date, this is the most comprehensive study characterizing RNA expression in OPSCCs. In this way, we have developed and independently validated a 60-gene signature for risk stratification of OPSCC patients.

HPV status has been shown to be a robust prognostic marker in OPSCC. The favorable prognosis of HPV+ patients leads to various deintensification strategies aiming to maintain high survival rates while reducing treatment toxicity [20]. Currently, this is an active area of clinical research, and multiple studies have demonstrated promising results from deintensification clinical trials for HPV+ OPSCC patients [21]. Despite favorable prognosis in general, there are approximately 20% of HPV+ OPSCC patients who died of disease [2,22]. It is hypothesized that there may be distinct genetic subtypes of HPV+ OPSCC, resulting in variable responses to therapy [23]. Thus, deintensification may not be appropriate for all HPV+ OPSCC patients. In our study, the 60-gene signature was robust at distinguishing high- and low-risk patients in the HPV+ patient group. Thus, our gene signature could contribute to ongoing trials by improving the selection of low-risk HPV+ OPSCC patients for deintensification treatment.

Multiple genes in the 60-gene signature were previously implicated in cancer progression. For example, LYN and MEI1 were included as favorable genes in the signature. LYN has the capacity to stabilize focal adhesion complexes and its overexpression were associated with better OS in acute myeloid leukemia [24,25]. Similarly, patients with higher expression levels of MEI1 have better OS than those with lower expression levels in cervical cancer [26]. Another gene, TRIB3, was an unfavorable gene in the signature. It has recently been identified as a stress sensor in response to various tumor microenvironments [27]. In addition, TRIB3 also shows a significant correlation with worse OS in colon cancer [28]. To further evaluate the functional roles of the genes in the signature, DAVID was used to identify gene oncology functional enrichment [29]. The most significantly enriched terms include regulation of glucose transport and inflammatory response. Previous studies indicate that glucose metabolism is a potential therapeutic target in cervical cancer, and high tumor inflammatory response predicts better survival in OPSCC [30,31]. Thus, the roles as well as the underlying mechanisms of these biomarker genes in OPSCC warrant further investigation.

In summary, we developed and validated a clinically feasible gene signature for OPSCC prognosis. The robust performance of the signature on multi-institutional OPSCC patients indicates its general applicability in clinical practice.

## Author contributions

XL led the bioinformatic and biostatistical data analysis. PL performed the RNA-seq experiments and updated the clinical data. XL and PL contributed equally to this study. R.D.C, K.A.LK, J.S.L, H.A.G and W.L.T provided the tumor specimens and patient clinical data. JL provided statistical guidance throughout the study. XW contributed to the study design and project supervision. All authors contributed to writing and reviewing the manuscript and approved the submitted version.

## Declaration of Competing Interest

The authors have no conflicts of interest to declare.

## Appendix Supplementary materials

Image, application 1
