# Supplementary material for: A prognostic gene expression signature for oropharyngeal squamous cell carcinoma
Source: eBioMedicine. 2020 Oct 7;61:102805. doi: 10.1016/j.ebiom.2020.102805 (PMC7648117; doi:10.1016/j.ebiom.2020.102805)
Supplement: Supplementary file 1 [file mmc1.pdf]

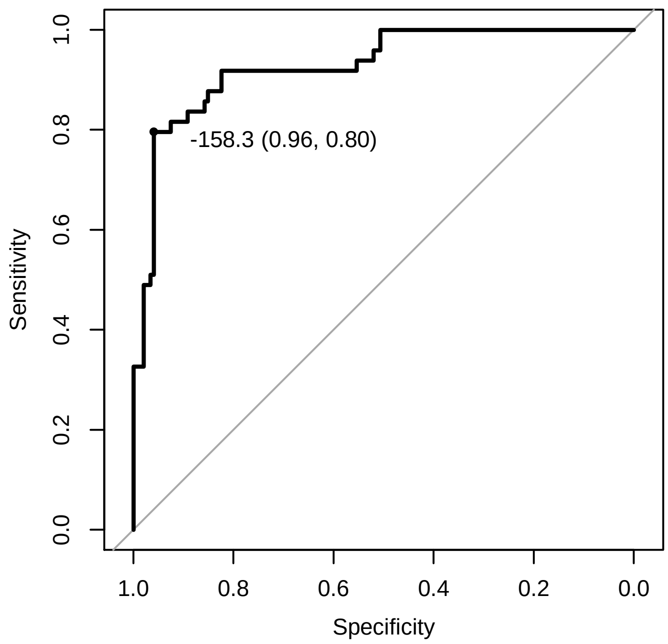

Supplementary Figure S1. ROC curve for 5-yr OS in the training cohort. The optimal cut-off value is shown.

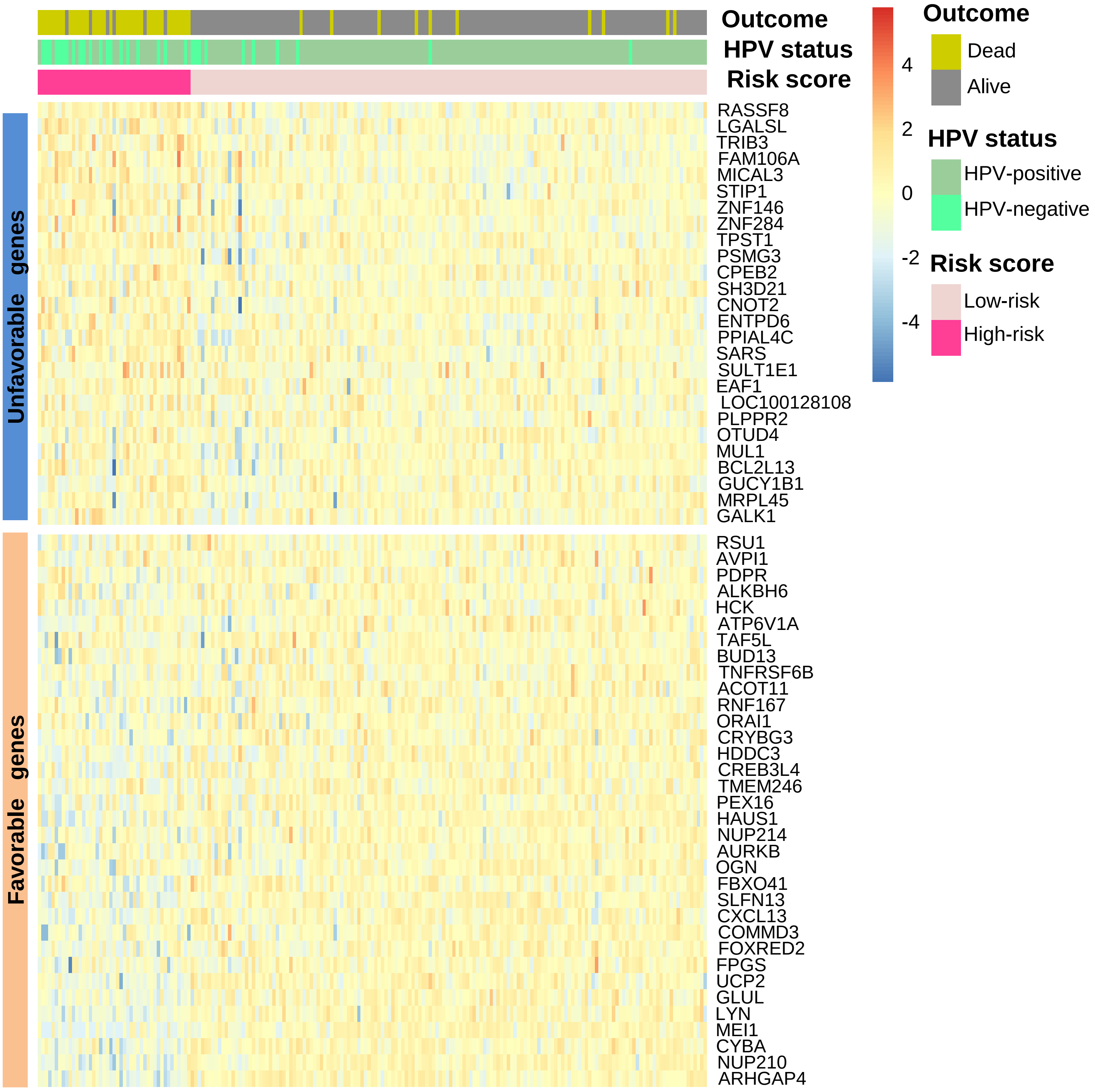

Supplementary Figure S2. Heatmap of the 60 genes in training cohort

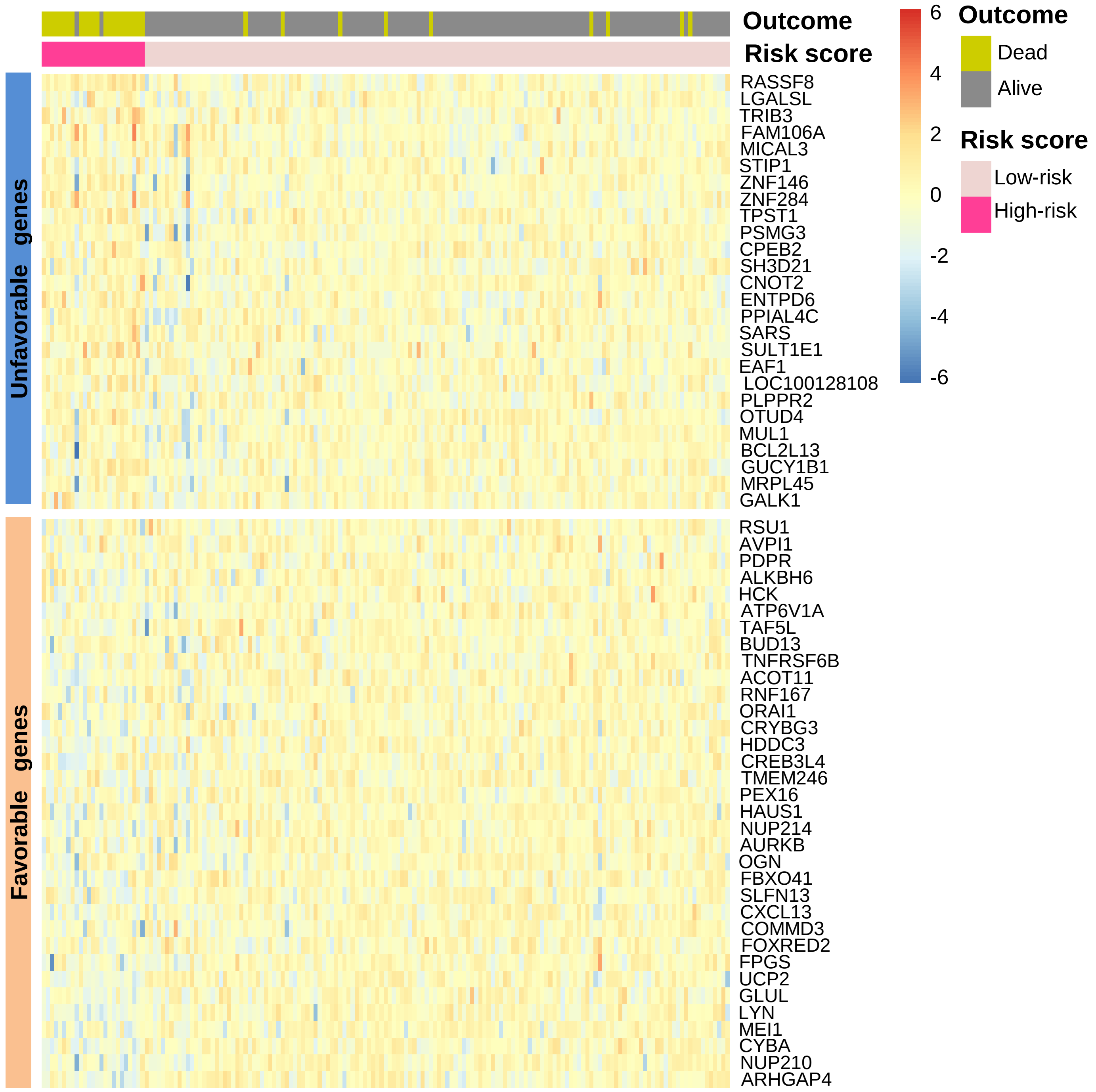

Supplementary Figure S3. Heatmap of the 60 genes in training HPV+ cohort.

Supplementary Table S1. The Frequency Count of the 77 Genes across 1000 Iterations

Gene symbol	Total frequency count
GLUL	700
MEI1	700
ARHGAP4	697
LYN	697
NUP210	697
CYBA	685
ACOT11	303
ALKBH6	303
AURKB	303
AVPI1	303
CNOT2	303
COMMD3	303
CPEB2	303
CXCL13	303
FAM106A	303
FBXO41	303
FOXRED2	303
FPGS	303
GALK1	303
GUCY1B1	303
LGALSL	303
MICAL3	303
MRPL45	303
OGN	303
ORAI1	303
OTUD4	303
PEX16	303
PPIAL4C	303
RSU1	303
SH3D21	303
SLFN13	303
TAF5L	303
TMEM246	303
TNFRSF6B	303
TPST1	303
TRIB3	303
ZNF146	303
ZNF284	302
NUP214	302
HCK	300
LOC100128108	300

ATP6V1A	298
CREB3L4	298
EAF1	298
RNF167	298
ENTPD6	295
MUL1	295
PSMG3	295
CRYBG3	292
UCP2	292
BCL2L13	284
HDDC3	284
SULT1E1	284
SARS	283
PDPR	265
PLPPR2	265
RASSF8	259
HAUS1	256
BUD13	228
STIP1	228
VEZF1	177
LPGAT1	175
CEACAM19	128
ARHGEF40	120
BSPRY	107
INTU	107
PTPRZ1	107
RCBTB1	107
TMEM120A	75
PELO	44
ARRDC4	38
RSL1D1	19
SPAG9	12
HERC5	11
IKBKG	11
CENPE	8
LRP6	5

Supplementary Table S2. The 60-Gene Signature.

Gene symbol	Univariate Cox *P* values	Univariate Cox z-scores	Multivariate Cox *P* values	Multivariate Cox z-scores
RASSF8	1.6E-04	3.77	1.0E-04	3.89
LGALSL	2.4E-04	3.67	1.9E-03	3.11
TRIB3	3.9E-04	3.55	3.6E-04	3.57
FAM106A	6.8E-04	3.40	9.8E-03	2.58
MICAL3	7.5E-04	3.37	7.6E-03	2.67
STIP1	8.6E-04	3.33	1.3E-03	3.21
ZNF146	1.6E-03	3.16	2.0E-03	3.10
ZNF284	1.9E-03	3.10	0.012	2.52
TPST1	4.1E-03	2.87	1.5E-03	3.17
PSMG3	4.6E-03	2.84	0.013	2.49
CPEB2	5.5E-03	2.78	3.6E-03	2.91
SH3D21	6.2E-03	2.74	0.017	2.38
CNOT2	8.7E-03	2.62	0.014	2.47
ENTPD6	9.4E-03	2.60	0.011	2.54
PPIAL4C	0.012	2.51	5.7E-03	2.76
SARS	0.013	2.49	0.012	2.53
SULT1E1	0.018	2.36	0.030	2.17
EAF1	0.021	2.30	0.019	2.34
LOC100128108	0.026	2.22	0.035	2.11
PLPPR2	0.027	2.21	0.037	2.08
OTUD4	0.028	2.20	8.3E-03	2.64
MUL1	0.036	2.10	0.032	2.15
BCL2L13	0.037	2.09	0.027	2.21
GUCY1B1	0.039	2.07	0.019	2.35
MRPL45	0.039	2.07	0.035	2.11
GALK1	0.041	2.05	0.028	2.20
RSU1	0.038	-2.08	0.032	-2.14
AVPI1	0.038	-2.08	0.046	-2.00
PDPR	0.035	-2.11	0.045	-2.00
ALKBH6	0.017	-2.39	9.3E-03	-2.60
HCK	0.015	-2.44	0.022	-2.29
ATP6V1A	0.013	-2.48	0.015	-2.42
TAF5L	9.8E-03	-2.58	2.8E-03	-2.99
BUD13	9.7E-03	-2.59	0.022	-2.29
TNFRSF6B	6.0E-03	-2.75	4.9E-03	-2.82
ACOT11	4.2E-03	-2.86	1.0E-03	-3.29
RNF167	3.6E-03	-2.91	8.1E-03	-2.65
ORAI1	3.5E-03	-2.92	5.2E-03	-2.80
CRYBG3	3.3E-03	-2.94	5.5E-04	-3.45

HDDC3	2.6E-03	-3.02	1.6E-03	-3.15
CREB3L4	1.8E-03	-3.12	1.6E-03	-3.16
TMEM246	1.5E-03	-3.18	1.7E-03	-3.14
PEX16	1.2E-03	-3.24	4.5E-03	-2.84
HAUS1	9.4E-04	-3.31	1.6E-03	-3.16
NUP214	6.1E-04	-3.43	1.5E-04	-3.80
AURKB	5.3E-04	-3.46	4.4E-04	-3.52
OGN	5.3E-04	-3.47	3.6E-04	-3.57
FBXO41	4.3E-04	-3.52	8.9E-05	-3.92
SLFN13	9.4E-05	-3.91	1.3E-04	-3.82
CXCL13	8.3E-05	-3.94	1.1E-03	-3.25
COMMD3	6.6E-05	-3.99	2.3E-04	-3.68
FOXRED2	3.5E-05	-4.14	5.8E-06	-4.53
FPGS	2.9E-05	-4.18	2.7E-04	-3.64
UCP2	5.6E-06	-4.54	1.2E-04	-3.85
GLUL	4.1E-06	-4.61	2.2E-05	-4.24
LYN	2.0E-06	-4.76	6.9E-06	-4.50
MEI1	4.7E-07	-5.04	2.6E-06	-4.70
CYBA	3.0E-07	-5.12	1.1E-05	-4.40
NUP210	1.3E-07	-5.28	3.1E-07	-5.12
ARHGAP4	3.9E-08	-5.49	2.7E-07	-5.14
